# Cases of the Extraction of the Cataract; with Practical Remarks

**Published:** 1791

**Authors:** Richard Sparrow

**Affiliations:** one of the Surgeons to the Charitable Infirmary, Dublin; and Member of the Royal College of Surgeons in Ireland.


### IV. *Cases of the Extraction of the Cataract; with practical Remarks. By Mr.* Richard Sparrow, *one of the Surgeons to the Charitable Infirmary, Dublin; and Member of the Royal College of Surgeons in Ireland. Communicated in a Letter to* William Lister, *M. D. Physician to Saint Thomas's Hospital in London, and by him to Dr.* Simmons.

### CASE I.

IN the month of October, 1788, I was consulted by the Rev. Mr. Johnson, Curate of Longford, for a complaint in his eyes, which he informed me had, for upwards of three years, impaired his sight so much as to disqualify him for performing divine service.

The account he gave me of his disease was, that, in the summer of 1785, after working in his garden till he was rather warm, he took off his wig, and laid a wet napkin on his head, (which it seems was a frequent custom of his); that some short time after he was attacked with severe pain in his head, which in four days was succeeded by so great a dimness of his sight, that he could not distinguish a letter in a book. Having

Having applied to fome country practitioners, whofe prefcriptions afforded him no relief, he came to Dublin, and there, at different times, put himfelf under the care of a phyfician and an oculift, both of whom miftaking his cafe for an affection of the optic nerve tending to gutta ferena, put him under various courfes of medicine, ordered him fometimes warm, fometimes cold bathing, large and frequent topical bleedings, blifters, fetons, &c., which had no other effect than that of impairing his general health.

It was not for a year and a half after the firft attack that the real nature of his complaint was difcovered by two phyficians of eminence in this city: they told him he had cataracts, and judicioufly advifed him to lay afide all farther ufe of medicine; but, from his advanced age, were, I believe, little inclined to recommend a furgical operation. Tired, at length, with his diftreffed fituation, he determined on feeking relief at the only fource from which it could poffibly be obtained — the hand of the furgeon, and with this intention he called upon me.

I examined his eyes, and perceived a cataract in each; that in the right eye perfectly opaque, the left not quite fo, but permitting very little useful vision. There was a remarkable relaxation of the tunica conjunctiva in both eyes, folding up near one half of the cornea, and looking like a tear in each eye. In other respects his eyes had that appearance which experience teaches us is favourable to the success of an operation, viz. the ball of the eye of the natural shape and fulness; the cornea transparent, and free from all opacity; the pupil of the natural size and shape, dilating and contracting freely; but, above all, the power of distinguishing any opaque body placed between the eye and the light, the only certain test of the sound state of the optic nerve.

His general health was now restored, nor was he subject to pain in any part of his head. My chief objection, therefore, was his advanced age, he being turned of sixty-five years. At my request a consultation was held, in which every objection to operation was laid before him; but such was his impatience to obtain a chance of restoration of sight, that he paid little attention to our objections, and declared his fixed determination to submit to an operation as soon as it could with propriety be attempted. He then went to the country, and returning in November, called again on me. The only preparation paration I thought neceffary was a gradual diminution of the ufe of animal food for a fortnight; and for about a week previoufly to the operation he was ordered to bathe his feet in warm water every night at bed time. It was his wifh to have only the right eye operated on, keeping the other in referve in cafe of failure of the firft.

As the extraction of the cataract is an operation confeffedly one of the niceft in its execution, and the moft important in its effects, that the chirurgical art can boaft of, I flatter myfelf that a detail of the different fteps of it will not be thought tedious in a cafe like the prefent, which has been attended with the moft complete fuccefs.

### *Method of Operating.*

For the operation a chamber was chofen into which only a moderate degree of light was admitted; as a bright light irritates the eye, makes it unfteady, and forces the pupil to contract too much.

On the 20th of November, 1788, I proceeded to operate in prefence of Dr. Clarke and fome other medical gentlemen of this city. Having placed the patient on a low chair, with his his right eye obliquely to the light, and being feated oppofite to him, the upper eyelid was raifed by my affiftant and friend, Mr. Richards, and whilft I depreffed the lower one with my right hand, I with my left paffed a knife, having a long and narrow point, through that part of the cornea next the external angle of the eye, within half a line of its junction with the fclerotica, and a little higher than the center of the pupil, and pufhing it forwards to the oppofite fide, made nearly a horizontal fection of full one half of the cornea. The aqueous humour immediately flowing out, the lids were clofed, and the eye fuffered to reft for a minute or two: the patient was then defired to open the eye, when, having introduced the inftrument recommended by De Wenzel to divide the capfule, the opaque cryftalline was pufhed out of the eye by the contraction of its mufcles, without the neceffity of any external preffure whatever on the globe. After gentle friction on the cornea to collect any fragments of the cataract in the pupil, nothing appearing, and the pupil feeming perfectly clear and round, the eye was clofed.

Having been in the habit of applying fome fedative application to the eye after this operation, tion, which, from the neceſſity of frequent renewal, I had found very inconvenient to the patient, in this caſe I made uſe of nothing more than a bit of fine ſoft lint, ſecured with a light compreſs and roller, and the patient was laid in bed on his back, with his head inclined to the left ſide. During the ſhort time the eye was ſuffered to remain open, after the operation, the patient could diſtinguiſh the panes of glaſs in the oppoſite window.

Upon examination of the cryſtalline, it was found of the natural ſize; its center of a very firm conſiſtence, and of a dark topaz colour, while the circumference, which was of a whitiſh colour, was ſoftened to the conſiſtence of jelly.

On the day of the operation, and the two ſucceeding ones, he was quite free from pain or uneaſineſs in the eye.

On the fourth day he could diſtinguiſh, through the bandage, when the window ſhutters of the chamber were opened or cloſed.

On the fifth day, the eye became more ſenſible, and watered a good deal; the lids ſtuck together, and gave ſome uneaſineſs: this probably aroſe from a ſevere change in the weather. He was adviſed to apply his ſaliva on the end of his finger finger to the edges of the eyelids when they adhered; and this ſimple application he always found removed the adheſion, and gave him eaſe.

On the ninth day, the irritation having ſubſided, I removed the bandage, and opened the eye. Some inflammation appeared on the lower part of the conjunctiva; the wound of the cornea was perfectly united; the eye naturally full; the pupil of the natural ſhape, ſize, and clearneſs: he could diſtinguiſh the different objects round the chamber, and could even ſee the hands on my watch. The eye was cloſed, and covered as before. He was allowed a more generous diet.

On the eleventh day, he could tell the hour by my watch.

On the ſeventeenth day I removed his bandage, and pinned a bit of green ſilk to his night-cap. The dilatation and contraction of the pupil were now nearly natural. He was ordered to bathe the eye with cold water.

On the twenty-fourth day, with the aſſiſtance of a proper glaſs, he could read a newſpaper, and in a week more returned to the country, his eye and viſion being then in the following ſtate:

The eye of the natural ſize and ſhape; the cicatrix of the cornea ſcarcely perceptible, and ſo cloſe to the ſclerotica as to give no impediment whatever to viſion; the pupil a little ſmaller than natural, but its ſhape, dilatation, and contraction perfect. With the aſſiſtance of a convex glaſs he could ſee to read the ſmalleſt print as well as he ever did in his life. The tunica conjunctiva, which had been ſo much relaxed previouſly to the operation, had now recovered its tone, probably from the degree of inflammation neceſſarily attending the operation.

In the courſe of two years, which have now elapſed ſince the operation, I have had many letters from this gentleman, and have often ſeen him in town. His ſight is as perfect, when aſſiſted by a proper glaſs, as it was at the age of twenty; and ſince a month after the operation he has conſtantly gone through the whole of his duty as a curate with as much eaſe to himſelf as if his ſight had never been affected.

### CASE II.

In the month of September, 1789, I was conſulted by Mr. Pollen, of Carlow, for a complaint in his eyes, which deprived him of all ufeful fight. Upon examination I found he had cataracts, which had been preceded by the ordinary fymptoms, gradually increafing from the firft attack, two years before, to that time, when he was fo blind as to be able to diftinguifh little more than light from darknefs. The appearance of his eyes and the colour of the cataracts were favourable for operation; but he was turned of fixty years of age, was very rheumatic, and, from a habit of drinking freely after dinner fince his fight became impaired, he had got a fulnefs and rednefs in his face which fhowed a confiderable determination to the head. Thefe circumftances, however, were by no means fufficient to forbid the operation where the appearance of the eyes was fo favourable; I therefore gave it as my opinion that his chance of reftoration to fight was confiderable. He immediately determined on the operation.

To obviate any inflammatory tendency he was put on a ftrict antiphlogiftic regimen for about ten days. I then extracted the opaque lens from the left eye by making an horizontal fection of the inferior half of the cornea, through which the cataract inftantly rufhed, accompanied, as nearly as I could guefs, by about half a tea-fpoonful of the vitreous humour. This accident was, I believe, occaſioned by a violent involuntary motion of the muſcles of the eye, for my aſſiſtant, Mr. Richards, made no preſſure whatever on the globe: the eye was cloſed, and ſuffered to reſt, to prevent any farther flow of the vitreous humour, which, notwithſtanding this precaution, took place in ſome degree. On opening the lids, the pupil appeared much dilated, but perfectly clear. I then cloſed the eye, and covering it with a bit of dry lint, a thin compreſs, and roller, laid the patient in bed, with his head inclined to the right ſide.

The cataract was the largeſt I had ſeen; it was of a dark brown colour, the edges, as uſual, being lighter in colour, and ſofter in conſiſtence.

Immediately after the operation, while I was taking ſome blood from him, he aſſured me that the wound of his arm gave him more pain than that of his eye.

On the day of the operation he had a ſenſation in the eye as if it wanted to open, a kind of vibratory feel, and, as he expreſſed it, frequent bright flaſhes of light ſeemed to come from his eye, ſo that he imagined candles were lighted in his chamber: he had alſo a flow of tears, tears, but unattended with heat or pain. Notwithſtanding theſe ſenſations, he ſlept perfectly well, and next day found his eye quite eaſy. The ſenſation of flaſhes of light returned at intervals.

On the third day he complained of no uneaſineſs, but ſtill perceived the flaſhes of light. I removed the bandage to try if any thing was amiſs. The eye, through the lids, appeared nearly as full as the other, and without opening it he could readily diſtinguiſh the different degrees of light admitted into his chamber for the purpoſe.

On the fourth and fifth days he was ordered to apply his ſaliva to the eyelids when they adhered or were uneaſy, which always gave him relief.

On the ſixth day he was permitted to get out of bed.

On the eighth day I opened the eye. The wound of the cornea was perfectly united; the eye ſeemed naturally full; the pupil clear, ſomewhat dilated, and of an oval form obliquely from ſide to ſide, the direction, I ſuppoſe, in which the cryſtalline had paſſed through it; with conſiderable turgeſcence of the veſſels of the tunica conjunctiva. He knew his wife and

 friends, friends, and could tell the colour of different objects. The eye was closed, and covered as before. Encouraged by these favourable circumstances, he, without my knowledge, indulged himself freely in the use of animal food and port wine, which, in a habit like his, kept up a degree of inflammation in the eye for some time, that made the application of a blister necessary. This soon removed all uneasiness, and his sight gradually became stronger.

On the twenty-fifth day from the operation, having got a glass which enabled him to read moderate-sized print, he returned to the country. I have had different letters from him since, and last summer I had an opportunity of examining his eye, as he came to town for some days. The pupil was a good deal dilated, still of rather an oval form, with very little power of contraction or dilatation, and that only at its superior part; but, notwithstanding this circumstance, his sight was so perfect, when assisted by a proper glass, as to enable him to read, write, and do all his usual business.

### CASE III.

In the month of October, 1789, James Kelly, a poor man, by trade a hatter, requested my affiftance for a complaint in his eyes, which rendered him incapable of earning his bread. He attributed his blindefs to the influenza, having been feverely attacked with that epidemic in the fummer of 1788; immediately after which he found his fight gradually diminifhing till about a month before he applied to me, when he became unable to diftinguifh objects. The fymptoms were the ordinary ones of cataract, and on examination I perceived one in each eye; the left completely opaque, and of a white colour, the other not quite fo, he being able to fee bright colours with it.

From the whitenefs of the left cataract I apprehended it was of a milky nature, being, I fuppofed, diffolved in the capfule; and from fome irregularity in the colour, at different points of it, I judged the capfule to be opaque. From this latter circumftance, notwithftanding the favourable appearance of the eye in other refpects, I had many doubts of the fuccefs of an operation; but he entreated me to give him even the chance that remained, as he could not be worse than in his present situation.

After the usual preparation, I proceeded to extract the left cataract, and having passed the knife through one side of the cornea, and approached the other, the eye turned towards the internal angle. This obliged me to pierce the opposite side as quickly as possible, which immediately gave me such command, that I turned the eye to the position I wished, and finished the section of the cornea. The aqueous humour flowed out, and from the extreme irritability of the eye the pupil instantly contracted to the size of a large pin's head, enclosing the cataract. I turned his back to the light, and, closing the eyelids, suffered him to rest for some minutes, but the pupil was so clearly contracted, that I had much difficulty in introducing the instrument recommended by De Wenzel to divide the capsule of the crystalline. After waiting, however, some minutes longer, the irritation subsided in some measure, and I effected it; the pupil became a little more dilated, and, by cautious pressure, I extracted a large portion of the crystalline in a broken and partly dissolved state; the remainder of it came away partly by pressure preffure and partly by the affiftance of a fmall hook.

I now was convinced of what I had before a fufpicion, that the capfule was partly opaque; but it feemed confined to a fmall portion of it, at the external part, which left full four-fifths of the pupil perfectly clear. From this circumftance, and the unavoidable lofs of fome of the vitreous humour during the operation, I thought it moft prudent not to attempt to extract the opaque capfule from an eye fo extremely irritable; I therefore clofed it, and having bled the patient freely from the arm, laid him in bed in the proper pofition, viz. on the oppofite fide to the eye operated on. This is a very neceffary precaution, as it tends to prevent ftaphyloma, or any dangerous flow of the vitreous humour.

From the great irritability of this patient's eye, I am perfuaded, that, had I made ufe of a fpeculum to fix it during the operation, the moft probable confequence would have been a fatal difcharge of the vitreous humour.

On the firft and fecond days after the operation he had a fenfation of flafhes of light before the eye, but without heat or pain. He took an opiate at night.

On

On the third day, the roller becoming loofe, I removed it: there appeared no inflammation of the eyelids: he could diftinguifh through them when I placed my hand between the eye and a candle. The eye was covered as before. From this to the eighth day he continued free from pain, and was allowed a more generous diet.

On the eighth day, having bathed the eye-lids with warm water, I opened them. The eye appeared fomewhat inflamed, but naturally full. The pupil was clear, except a very fmall portion of it next the external angle of the eye, which was obftructed by the opacity of the capfule at that part. The fhape of the pupil was fomewhat irregular, and larger than natural. He knew his wife's face, and thofe of fome friends who were in the chamber. The eye was covered as before.

On the fourteenth day I obferved a fubftance like mucus on the inferior part of the cornea near the cicatrix of the wound. I had feen this on the eighth day, but did not then take much notice of it. This day I examined it more attentively, and found it to be a fubftance like a very fine membrane in a floughy ftate, taking its rife from the cicatrix of the cornea. It was removed with a pair of fmall forceps, and its tenacity city was found to be confiderable : his fight was immediately improved by this little operation. The eye having watered freely for fome days before, he had been ordered a faturnine collyrium, but, as ufual in thefe cafes, with no effect.

As fome degree of inflammation was ftill prefent, which might be increafed by the application of the inftrument to remove this opaque fubftance, it was thought advifable to order a blifter to the back of the patient's neck, which in fome days had the defired effect.

On the twenty third day from the operation he could fee objects very diftinctly ; knew perfectly all his friends with the naked eye ; and as he walked through the ftreets he could readily read the names, &c. of the fhopkeepers written over their doors.

I intended purchafing a proper glafs for this man ; but he foon relapfed into a habit of drinking fpirituous liquors, to which he had formerly been much addicted, and knowing that I highly difapproved of this conduct, he was, I believe, afhamed to let me fee him.

I have at different times fince, and fo lately as a week ago, endeavoured to find him out, but without fuccefs : I have learned, however, from his friends that his fight is fo good as to enable enable him to work at his trade without the afſiſtance of a glaſs, but that he has led, and ſtill leads, a very profligate life.

It may be aſked what was the nature of the ſubſtance encloſed in the cicatrix of the cornea? As it had neither the appearance, nor the conſiſtence of a fragment of the cryſtalline lens, but rather that of a membrane, as already deſcribed, I ſuſpect it to have been a portion of the membrane of the aqueous humour, frequently deſcribed by De Wenzel as forming one ſpecies of ſtaphyloma after this operation; in which caſe it appears like a drop of limpid water reſting on the cicatrix of the cornea; but that it has ſome kind of covering we know from its not being removed by the motion of the eyelids. A caſe of this kind I have ſeen; the little ſac was removed by a ſnip of a pair of ſciſſars, but returned again next day. Its radical cure was effected by the natural motion of the eyelids.

In the preſent caſe, this membrane, inſtead of forming a little hernia containing aqueous humour, protruded between the edges of the divided cornea, and theſe fell into a ſloughy ſtate, giving the appearance already deſcribed.

CASE

### CASE IV.

In the month of July, 1790, John Hannan, a poor ſchoolmaſter from Kilkenny, aged forty-three years, applied to me for relief for blindneſs occaſioned by cataracts. Fifteen years before, an oculiſt, in this city, attempted to depreſs that of the left eye, in which he failed, and the man remained blind of it.

The cataract of the right eye had been gradually affected in the uſual manner for near ſix years, and at the time he came under my care all uſeful ſight had been loſt for two years. It was of an iron colour, and from a certain ſtreaked appearance on its ſurface I judged the capſule of the lens to be opaque. In other reſpects the eye looked well; the dilatation and contraction of the pupil were perfect, and I had reaſon to believe the optic nerve to be in a ſound ſtate: I, however, acquainted him with my doubts of ſucceſs from the opacity of the capſule; but his ſituation was ſo miſerably dependent, that he begged I would give him whatever chance remained.

When I undertook this operation I intended to have removed, with a pair of fine forceps, the the anterior part of the capſule, ſhould it be found in a ſtate of opacity, a thing very practicable in many caſes; but from the circumſtance I am about to mention I was obliged to alter my intentions. My aſſiſtant, Mr. Richards, ſecured the upper eyelid without making any preſſure on the globe: I introduced the knife into the cornea at the uſual place; but ſcarce had I done ſo when the eye (which was extremely irritable) ſuddenly turned in towards the noſe: this obliged me to uſe all poſſible diſpatch in piercing the cornea at the oppoſite ſide, and finiſhing the inciſion, to avoid being engaged in the iris, and which with conſiderable difficulty I effected; for ſo rapid and violent were the motions of the eye, that the blade of the knife, while paſſing through the cornea, was ſo much bent, that on reſuming its ſhape it made a noiſe that could be heard at the fartheſt part of the chamber. The conſequence of all this was, too ſmall an inciſion of the cornea, the great, and, I believe, moſt frequent cauſe of the failure of this operation in the hands of practitioners in general.

The pupil contracted over the cataract, and I had to wait full a quarter of an hour before I could introduce an inſtrument to divide the capſule, capſule, which appeared very opaque, and gave ſome reſiſtance to the needle; at length, however, it was effected, and by the cautious preſſure of my aſſiſtant I was enabled to ſeize the cataract with a ſmall hook, and extract it ſafely and entirely. It was large, of a brown colour, and hard conſiſtence. The capſule now appeared obſtructing more than half the pupil; but the quantity of vitreous humour loſt during the operation, the extreme irritability of the eye, and, above all, the ſmallneſs of the wound of the cornea, made it impoſſible for me to attempt the extraction of this opaque ſubſtance without incurring the greateſt hazard of deſtroying the eye. Having, therefore, puſhed the capſule as much to one ſide the pupil as I could with a ſmall blunt inſtrument, I cloſed the eye, and truſted to nature for the reſt.

I took ſome blood from his arm, and laid him in bed in the uſual poſition. At night he had an opiate. Next day he was free from all pain or uneaſineſs, feeling nothing more than a degree of warmth in the eye.

On the night of the ſecond day his bandage looſened and came off, and ſo little ſoreneſs had he in the eye, that, forgetting his ſituation, he rubbed and opened it. When I ſaw him next day day I found the eyelids without inflammation, and curiofity induced me to open them and examine the eye at this early period. The wound of the cornea feemed to be perfectly united, and the eye naturally full; but there appeared to be a good deal of inflammation and intolerance of light; the pupil was almoft entirely obftructed by the opaque capfule, and the interftices had a muddy appearance. As the eye watered much from the irritation of the light, I clofed it, with little hope indeed of his getting fight.

From this early opening of the eye it was for fome fucceeding days in a ftate of confiderable irritability, giving fome uneafinefs, and watering freely, but unattended with pain. I laid a blifter on the back of his neck, and kept up a difcharge for fome time, which had the defired effect.

I did not open the eye again till the fourteenth day. There was ftill fome degree of inflammation and turgefcence of the veffels of the tunica conjunctiva; the pupil had fome clear points towards the inferior and internal parts; he could diftinguifh my features tolerably well, the colours of a painted chair, &c. The wound of the cornea was flightly opaque. He had been been out of bed every day for a week paſt. His room was darkened.

On the 24th day, his ſight was improving faſt. Though more than 4-5ths of the pupil were obſtructed by the capſule, he could, through the clear points, diſtinguiſh colours accurately; knew his daughter's face, and with the aſſiſtance of a glaſs of conſiderable magnifying powers, he could read very large print. He had that imperfection of viſion, however, which is common after this operation for a ſhort time, viz. of ſeeing a candle and other objects double. For ſome days paſt he had walked out and waſhed the eye with cold water.

At the end of five weeks two of the clear points of the pupil were obſtructed by the capſule becoming opaque. The one next the internal angle of the eye ſtill remained clear, and though it did not exceed in ſize the head of a large pin, yet through it, with the aſſiſtance of a proper glaſs, he could ſee to read print of the ordinary ſize, to know his friends perfectly, and to do all the common offices of life with eaſe. He returned in this ſtate to Kilkenny. I have twice ſeen him in town ſince, and examined the ſtate of his eye. The clear point had increaſed ſomewhat in ſize, and his viſion was conſiderably improved.

From the event of this caſe we learn how very ſmall a portion of the pupil will admit a ſufficient number of the rays of light to afford tolerable viſion; but I am aware that many may be of opinion that the involuntary motions of the eye during the operation, and the conſequent difficulties, might have been prevented by the uſe of a proper ſpeculum oculi. To this I can only ſay, that from my own experience, as well as from that of others, I am inclined to believe that it would not have had that effect; and that when the eye is, either naturally, or from any other cauſes, in a very irritable ſtate, the uſe of an inſtrument of that nature only ſerves to increaſe the danger of an operation, as it evidently tends, by its preſſure on the vitreous humour, to render the iris more convex, and to throw it more in the way of the knife. But at the ſame time that it increaſes the hazard in this particular, it deprives the operator of the only means of obviating it; for it is a curious fact mentioned by De Wenzel, and of the truth of which I am convinced by my own experience, and that of others, that when the knife becomes engaged in the iris, the only ſafe method of extricating it is by making gentle friction on the cornea with a finger of the hand employed to depreſs the lower eyelid, by which means the iris iris is found to retract ſufficiently to give the operator an opportunity of finiſhing the ſection of the cornea. Now if the hand be employed in holding a ſpeculum, it is evident that this very important aid cannot be obtained. But ſhould even this accident be avoided by the dexterity of the operator, ſtill there will be, from the ſame preſſure, great hazard of a ſudden and deſtructive flow of the vitreous humour as ſoon as the inciſion of the cornea is finiſhed; at leaſt it will require more dexterity to avoid it than falls to the ſhare of operators in general. I am, therefore, of opinion, that, in diſagreeable caſes of this nature, the ſucceſs of the operation muſt always depend chiefly upon the dexterity of the operator, and that the uſe of a ſpeculum will only ſerve to increaſe his embarraſſment and the patient's danger.

*Practical Remarks.*

Having now finiſhed a faithful, though perhaps tedious, detail of theſe caſes, I ſhall, from them, and ſome others which have fallen under my obſervation, beg leave to ſuggeſt the following practical remarks:

Firſt, That the ſafeſt and beſt method of ſecuring the eye, during the operation, is to have the upper eyelid drawn up by an aſſiſtant, while the operator himſelf depreſſes the lower one, without making any preſſure whatever on the globe of the eye. In ordinary caſes I have ſhewn that a ſpeculum oculi is at beſt unneceſſary; and in the caſe of an irritable eye I have ſhewn that its uſe would be attended with embarraſſment to the operator and hazard to the patient.

Secondly, That a knife, with the point long and narrow, traverſes the anterior chamber of the eye with more eaſe than that in common uſe, (by which the eye becomes fixed) and from the increaſing breadth of the blade towards the handle the operator is enabled, by ſimply puſhing it forwards, to make a ſection of full one half of the cornea, a leading ſtep in the operation, and which always renders the ſubſequent one of extraction more ſafe and eaſy.

Thirdly, That, after ſuch a ſection of the cornea as is above deſcribed, the various ſedative and other applications commonly made uſe of to the eye, with a view to prevent inflammation, are altogether unneceſſary; and that the adheſion of the eyelids, which often takes place, is moſt eaſily and effectually removed by the ſaliva of the patient applied on the end of his finger, which can be done without removing the bandage, or danger of hurting the eye, as the patient's feelings will teach him to avoid giving pain by any degree of preſſure.

Fourthly,

Fourthly, That the loſs of any of the vitreous humour, during the operation, ſhould, if poſſible, be avoided, as its preſervation haſtens the recovery of viſion, (ſee Caſe I.) ; but that even a large portion of it may eſcape without material injury. It appears to me that the recovery of viſion is ſlow in proportion to the quantity of this humour that eſcapes, but that the loſs of it has generally the effect of preventing after pain and inflammation.

Fifthly, That the opacity of the capſule of the cryſtalline humour is always to be feared, as leſſening the chance of ſucceſs from operation ; yet that even under circumſtances forbidding its removal the patient may be reſtored to ſight, as it appears that a very ſmall portion of the pupil remaining clear is ſufficient for this purpoſe—(ſee Caſe IV.)

Sixthly, That a ſenſation of flaſhes of light coming from the eye the firſt days after the operation, when unattended with pain, is not an unfavourable ſymptom—(ſee Caſes II. and III.)

Seventhly, That a preternatural dilatation, irregularity, and loſs of power of motion in the pupil, after this operation, are no material impediments to viſion—(ſee Caſes II. and III.)

Eighthly, That though a temperate ſeaſon of the year is moſt deſirable for a patient undergoing this operation, yet that with proper precautions, neither the rigours of winter, nor the heats of ſummer, prevent its ſucceſs.

And ninthly, That when the conſtitution in general, and all parts of the eye, except the cryſtalline and its capſule, are in a ſound ſtate, even an advanced age is no bar to the ſucceſs of this operation; but that a very vitiated habit, even in youth, will prevent ſucceſs I know from diſagreeable experience: having a few years ago extracted cataracts from a young woman, who was extremely emaciated from amenorrhœa and other cauſes, the conſequence was, that the wounds of the cornea did not unite for many weeks, and not till after one eye had ſuppurated, though unattended with pain. I was induced to operate in this caſe from the very favourable appearance of the eyes, and as no accident occurred during the operation, and it was not ſucceeded by pain, or other circumſtances marking local injury, I think I may fairly attribute its failure to the badneſs of the patient's habit.

It may not be amiſs here to mention the preſent ſtate of Abigail Cremor's eyes, (the woman whoſe caſe is given in Vol. IX. Part II. of the London Medical Journal, and who had a cataract extracted from one eye, and depreſſed in

3 the the other, in the month of October, 1787.) I have ſeen her very lately, and her eyes and viſion are now in the following ſtate :—The pupil of the couched eye has its ſhape, dilatation, and contraction, perfect : it is, however, ſomewhat ſmaller than natural ; and though there is no apparent impediment to viſion, yet ſhe does not ſee objects ſo diſtinctly with that eye as with the other, in which the pupil is larger than natural, in ſome degree irregular, and without any power of motion, but viſion is as perfect as it is ever found to be after the removal of the cryſtalline from its natural ſituation.

I had long obſerved a circumſtance in this woman's caſe which puzzled me exceedingly; it was, that ſhe could not diſtinguiſh a letter in a book, even when aſſiſted by the beſt glaſſes, though her ſight was ſuch as to enable her, with the naked eye, to ſew and make all her own cloaths, to know the hour by a clock, and, in ſhort, to do moſt of the offices of life with perfect eaſe. I had often expreſſed my ſurpriſe at this peculiarity; but it was not till laſt ſummer that, with much ſeeming reluctance and mortification, ſhe confeſſed that ſhe had never learned to read, and that ſhe was unacquainted even with the letters of the alphabet : thus had I an explanation of a circumſtance which, from the appearance of this woman's eyes, and her power of viſion in other reſpects, had led me to form various conjectures relative to her peculiarity of viſion; but which, from my ignorance of the real cauſe, appeared altogether inexplicable.

Having in the hiſtory of the preceding caſes ſometimes mentioned the name of De Wenzel, I think it neceſſary to add, that his method of operating, together with a number of curious and intereſting particulars relative to the extraction of the cataract, are to be found in a moſt excellent practical treatiſe, entitled *Traité de la Cataracte, avec des Obſervations*, &c., publiſhed by his ſon at Paris in 1786, in one volume, octavo. His inſtruments are delineated in a plate at the beginning of the book. A good deal of experience in this branch of ſurgery had confirmed me in my favourable opinion of that work, and it appeared to me to contain information of ſuch public utility, that I had determined on an Engliſh tranſlation of it, with caſes and remarks, illuſtrating the juſtneſs of the obſervations in general; but at the ſame time pointing out ſome particulars in which experience had taught me that implicit faith was not to be yielded even to ſuch authority. On making the neceſſary inquiries previous to this undertaking, I found I had been anticipated in the tranſlation by Mr. Ware, of London, who had already already made confiderable progrefs in it; and from the profeffional character of this gentleman it is not to be doubted that every juftice will be done both to the Author and the Public.

*Dublin,*

March 8th, 1791.

